# The role of disasters in shaping narratives of resilience and transformation in Puerto Rico

**DOI:** 10.1016/j.crsust.2023.100227

**Published:** 2023-07-13

**Authors:** Alaina D. Kinol, Laura Kuhl

**Affiliations:** School of Public Policy and Urban Affairs, Northeastern University, 310 Renaissance Park, Boston, MA 02115, United States

**Keywords:** Energy transitions, Transformational policy change, Climate resilience, Hurricane Maria, Puerto Rico, Disasters

## Abstract

Extreme weather events can act as “focusing events” that open windows of opportunity in the policy process for increasing resilience and transforming existing systems to be more sustainable and just. However, due to the multiple and contested meanings of resilience, it is uncertain to what extent a focusing event will foster transformational policy change as opposed to re-entrenching existing systems and structures. We conducted quantitative content and qualitative narrative analyses of Puerto Rican climate and energy policy before and after Hurricane Maria to assess the effect of a climate-induced disaster on the framings of resilience and transformation. We find that these terms are used predominantly in service of changes needed to promote the stability of the existing energy system. This suggests that after Hurricane Maria, achieving stability has been the dominant goal for resilience and transformation. As long as those responsible for the Puerto Rican energy system emphasize stability without actively working to enable longer-term transformational change, Puerto Ricans are unlikely to experience a rapid transition toward a sustainable, inclusive energy system.

## 1. Introduction

Increasing use of the terms resilience and transformation in social and environmental policy, particularly energy policy, reflects a growing acknowledgement of the need for clean energy transitions that also strengthen communities' resilience to disasters and other climate-induced shocks and stresses. While there is a dramatic rise of these terms on the policy agenda globally, it is widely acknowledged that the meanings of these terms are contested. Policy narratives are constructed by actors in the policy process to frame problems and solutions in particular ways and policymakers use concepts including resilience and transformation strategically in support of diverse goals (Bevan, 2020; Shanahan et al., 2018). As we seek to better understand processes of energy transitions, it is critical to examine policy narratives to better understand how these contested concepts are employed in service of diverse visions of the future and how external events can shape these narratives.

Resilience and transformation can be deployed to attain a range of ends, but disasters may cause a shift in which of these narratives are dominant and to what they apply (Kuhl, 2021a). Without a critical lens examining the ways transformation and resilience are embedded in policy discourse, the potential of climate and energy policies to address existing inequities among relevant actors and systems may be missed. Similarly, whether post-disaster policy narratives of resilience lead to transformational change or even reduce vulnerability to future climate disruptions – especially for those most at risk – is a question worthy of further investigation.

Puerto Rico's experience of Hurricane Maria in 2017 provides an ideal case study to examine the strategic use of resilience and transformation narratives, as well as the role of climate disasters as focusing events for energy transitions. Hurricane Maria was a catastrophic category four hurricane that made direct landfall in Puerto Rico (Keellings and Ayala, 2019). An estimated 2975 human lives were lost in the hurricane, which was the third most expensive storm in U.S. history, costing $100 billion (Goodell, 2018). Hurricane Maria created a health crisis and humanitarian disaster when it demolished more than 300,000 homes, left nearly the entire population of 3 million people across Puerto Rico without power, and cut off access to food and medical services for weeks, especially for already vulnerable and marginalized populations (Mickelson, 2019; Sotolongo et al., 2021). The collapse of the electric system revealed deep structural problems in Puerto Rico, particularly related to lack of investments in infrastructure, poor governance and finance, and opened up conversations on the need for equitable energy transitions and the importance of addressing underlying vulnerabilities (De Onis, 2021; Perez-Lugo et al., 2021; Santiago et al., 2020). Hurricane Maria was not an isolated disaster; it was compounded by the existing debt crisis and resulting austerity measures. Subsequent disasters, including 2019 earthquakes, COVID-19, and Hurricane Fiona in 2022 have reinforced these challenges, but Hurricane Maria remains a critical juncture point for understanding climate and energy policy in Puerto Rico.

This paper examines how resilience and transformation narratives are employed in climate and energy policy and the changes in these narratives after a disaster. Specifically, we address two research questions: 1) How are resilience and transformation employed in Puerto Rican policy narratives relating to climate and energy policy? 2) How did the salience and framing of resilience and transformation change after Hurricane Maria and what does this reveal about the role of disasters in shaping climate and energy policy? Using textual analysis of a dataset of Puerto Rican climate and energy policies from 2008 to 2021, we characterize narratives of resilience and transformation and compare narratives in policies pre- and post-Hurricane Maria.

This study contributes to scholarship on sustainability transformations to better understand leverage points, resilience, and vulnerabilities in complex systems in several ways. First, analyzing the diverse conceptualizations of resilience and transformation in climate and energy policy provides insights into the contested nature of these concepts in practice and the potential tradeoffs that they may reflect. Second, this research contributes to our understanding of the ways climate-induced extreme events may prompt transformational change or reinforce the status quo. This opens further avenues of research to understand the potential opportunities for climate-induced extreme events to enable climate and energy policy, as well as the dangers of climate-induced extreme events for sustainable transitions, including transitions in energy systems (Béné et al., 2018b; Gillard et al., 2016; Köhler et al., 2019).

## 2. Resilience, transformation, and disasters in climate and energy policy

### 2.1. Contested narratives of resilience

Increasing resilience has emerged as a clear goal for climate and energy policy, particularly in post-disaster contexts. Understanding the ways resilience is framed is important because of its potential to support transformational change (Béné et al., 2018a), but also the risk that resilience narratives may support the maintenance of existing systems and structures if politics and inequities are inadequately accounted for (Folke et al., 2010; Olsson et al., 2014). Both stability and change are conceptualized as components of resilience (Béné et al., 2018a; Folke et al., 2010; Folke et al., 2003; Leach et al., 2012; Olsson et al., 2014). Consequently, resilience narratives can be used to describe a wide range of policy goals and can be strategically deployed by policymakers in support of these potentially competing goals. The socio-ecological systems (SES) literature describes resilience as an ability to respond to systemic disturbances with some degree of stability (Folke et al., 2010; Gallopín, 2006). Reflecting its foundations in disaster risk reduction, resilience is often described as the capacity to ‘build back better’ (Carr, 2019), a somewhat contradictory conceptualization that acknowledges the need for change but maintains a focus on a return to previous conditions. Such definitions have been critiqued for suppressing a critical analysis of what is built back, for whom it is better, and who gets to choose (Carr, 2019; Ensor et al., 2018; Eriksen et al., 2015; Jones et al., 2020.

Critical social science scholars have long questioned the desirability of social system persistence in the context of social inequity and power imbalances (Adger, 2000; Baker, 2019; Jones et al., 2020; Welsh, 2014), especially when there is a potential for maladaptive outcomes or unintended negative impacts (Eriksen et al., 2021; Kuhl, 2021b; Magnan et al., 2016). While some have critiqued resilience narratives as “nihilistic” acceptance of exposure and vulnerability to danger (Evans and Reid, 2013), other scholars argue that the concept embeds a “neoliberal” emphasis on individual responsibility and self-reliance into governance, creating limited, shallow effects for stakeholders (Evans and Reid, 2013; Joseph, 2013). By offering narrow possibilities for change before a disaster, resilience has been critiqued as falsely “creat[ing] stakeholders in need of empowerment” (Grove, 2014). This has led to debate within the literature regarding whether communities and networks serve as needed sources of enhanced resilience and recovery (Aldrich, 2018; Sou, 2019; Zebrowski and Sage, 2017) or as corrupt and exclusive centers of disaster capitalism that prevent resilience (Imperiale and Vanclay, 2020).

In contrast to conceptualizations of resilience that focus on stability and rebuilding, resilience can also be characterized as part of adaptation, suggesting a stronger emphasis on change (Folke et al., 2010; Gallopín, 2006; Magnan et al., 2016). Attention to change has always been present in resilience scholarship, with foundational literature addressing the importance of adaptive cycles and panarchy (Gunderson and Holling, 2002). Some scholars explicitly argue that “bouncing back” is a misrepresentation of resilience, and that resilience is better understood as the ability to manage change and stay away from or avoid dangerous thresholds and tipping points (Walker, 2020). From this perspective, resilience can contribute to transformation, as transformation at one scale may require maintaining resilience at other scales (Walker, 2020). However, beyond having a larger scope than incremental change, and being path-shifting, multi-scalar, and systemic (Fedele et al., 2019), there are no clear metrics to determine at what point social change should be considered “transformational” or to identify what causes this change (Kuhl et al., 2021). Outcomes of transformation and the processes that lead to them are inherently uncertain, unpredictable, and contingent on human relationships to each other and the natural world (Nightingale et al., 2021).

Historically, change in energy systems has been remarkably slow, but with the growing awareness of the necessity of rapid transitions to clean energy, scholars, practitioners, and policymakers question how to enable transformations of a scale and speed that has never been achieved (Markard et al., 2020; Newell and Simms, 2021). Energy infrastructure is widely understood to be durable and path dependent and subject to lock-in (Fouquet, 2016; Unruh, 2000). In other words, existing systems are remarkably persistent, but this very persistence undermines the ability of these systems to transformationally respond to change. Scholarship on transitions shows that transformation has happened only when pressure for change aligns at multiple levels (Geels et al., 2017; Sovacool, 2016). Transformations in energy systems are generally theorized to result from either bottom-up or multi-system alignment, with change occurring when goals align across local, institutional, and socio-technical landscapes (Bergek et al., 2008; Rip and Kemp, 1998). This process is complicated however, by the reality that there are competing views on desired transformation outcomes even among actors responsible for designing public policy (Köhler et al., 2019; Kooiman, 2003; Shove and Walker, 2007). Understanding the conditions under which rapid change is possible is urgently needed (Köhler et al., 2019), as is understanding why such transitions do not occur or fail in practice (Berkhout et al., 2004).

Additionally, the nature of the relationship between transformation and sustainable transitions is contested. While some authors have framed transition and transformation as two distinct processes (Markard et al., 2020), others have described transformation as the result of a transition (Patterson et al., 2017), or transition as the outcome of transformations in sustainability regimes (Kuhl et al., 2021; Smith et al., 2005). Both can be characterized as a response to a normative desire for change from unsustainable systems using political and technological innovation and with the goal of building adaptive capacity (Foxon et al., 2009; Hölscher et al., 2018). Transitions have often been framed as sub-systemic, sector-level changes, such as in the energy sector, in which technology-driven efforts that follow the trajectory of a context-specific ‘transition pathway’ are favored (Berkhout et al., 2004; Patterson et al., 2017) and collectively these transitions lead to broader transformations. The transitions literature recognizes the concept as both social and technical, and more recently has emphasized social innovation and justice (i.e., just transitions) as important components of transitions in addition to the stronger historical emphasis on technological innovation (Carley and Konisky, 2020; Newell and Mulvaney, 2013). Transitions and transformation emerge from different academic transitions and scholars debate the relationship between them. However, in our analysis we analyze the terms jointly, as both concepts ultimately underscore the urgency of change beyond incremental progress toward coordinated social and ecological sustainability, and are used in practice to reference similar processes.

### 2.2. The role of disasters in energy transitions

Major disruptions can create opportunities for policy change, called policy windows in the public policy literature, by interrupting the power dynamics associated with existing systems (Birkland, 1997; Ellis, 2020; Kuhl, 2021b; O'Donovan, 2017). As the frequency and extent of climate-related disasters increase, the impact of these events on energy transitions is increasingly relevant (Kuhl, 2021a). During moments of disruption, the politics of sociotechnical systems become open to reconsideration, redesign, and reconfiguration (Ellis, 2020; Graham, 2010). Policymakers and other actors can take advantage of the policy window created by climate-induced disasters to introduce resilience as a salient frame for their policy objectives (Birkmann et al., 2010; Friedman et al., 2019; Jetten et al., 2021; Kuhl, 2021b; McSweeney and Coomes, 2011; Moatty et al., 2021). Despite literature suggesting that disasters may create windows of opportunity, much less attention has been paid to the ways that policy narratives can be employed to shape transformational change, including the *directionality* of change, or whether this change will be equitable. Commitments to equity alone do not guarantee that sociotechnical transformations will be inclusive or equitable; power is deeply embedded in existing systems and entrenched interests are resistant to change (Ellis, 2020; Feola, 2015; Jones et al., 2020; Patterson et al., 2017; Stephens, 2020). The violence of war and revolutions illustrate the resistance that often accompanies transitions and the potential for rapid transitions to reinforce inequality (Newell and Simms, 2021). Because policy narratives can be selectively deployed to promote diverse policy objectives, these narratives are both indicative of the negotiated priorities of key actors in shaping transitions, as well as part of the enabling environment for these processes.

Resilience narratives often rise on the policy agenda after disasters where ‘building back’, ideally with additional adaptive capacity to prevent recurring or compounding crises and to address systemic vulnerabilities, becomes a policy priority (Jones et al., 2020; Matyas and Pelling, 2015). Disasters also reveal the underlying structural vulnerabilities that hinder resilience (Baker, 2019; Sotolongo et al., 2021), presenting a strong case for investments in resilience to address past injustices. Extreme weather events may be focusing events that drive change by creating “windows of opportunity” that focus the policy agenda to address vulnerability to climate change and the need for systemic or transformational change (Agrawal, 2011; Blanco and León, 2017; Kates et al., 2012; Kingdon, 1984). At the same time, the need to respond quickly to disaster may lead to policy approaches that provide a short-term response but with consequences that inhibit or undermine transformation in the long-term. In the absence of deliberate planning, resilience interventions have broadly been critiqued as having the potential to produce maladaptive outcomes or entrench harmful systems, e.g., “technological lock-in”, that could create hazards, increase individuals' or communities' vulnerability to future climate change, and limit future adaptive capacity (Atteridge and Remling, 2018; Barnett and O'Neill, 2010; Felgenhauer, 2015; Pelling and Manuel-Navarrete, 2011). The rigidity and durability of existing physical energy infrastructure creates technological lock-in, and perpetuates status quo power relationships and social norms that reinforce socio-political systems by limiting possibilities for visions of the future (Burke and Stephens, 2017). While exogenous forces such as crises can create opportunities to overcome this path dependence, escaping lock-in is not guaranteed (Unruh, 2002). Despite the occurrence of the window of opportunity and awareness of likely future disasters due to climate change, long-term resilience and a transition to clean, sustainable energy are not guaranteed after disaster.

Closely related to understanding how disasters create moments of opportunity, scholars have analyzed the ways that emergency frames have been both reactionarily and strategically deployed in climate policy to focus attention and underpin the prioritization, especially by governments, of climate action (Hodder and Martin, 2009; Patterson et al., 2021). Issue framing is grounded in cognitive psychology and refers to how public opinion or understanding of an issue is substantially affected by how that issue is presented: all communications frame the information they relay (Nisbet, 2009). Because they are often not consciously perceived and anticipate intuitive associations, frames have been very effective in shaping public opinion, especially for those without strong existing perspectives (Chong and Druckman, 2007). A growing evidence base suggests that framing climate change as an emergency may provide a disaster prevention, planning, and management foundation that unifies policymakers and increases the legitimacy of policy intervention (Davidson et al., 2020; Schneider, 2011). Frames that focus more on local vulnerabilities to climate change rather than on more global or economic impacts are more effective in increasing policy support than those that do not (Wiest et al., 2015), however this does not necessarily result in more effective climate policy outcomes.

Despite their potential effectiveness, emergency or crisis framing may have unintended consequences, such as legitimizing extreme and untested responses and less democratic, more exclusive decision-making (Patterson et al., 2021). This may result in entrenchment of the status quo or a “critical juncture” wherein existing power dynamics are challenged (Novalia and Malekpour, 2020; Pelling and Dill, 2010). Evidence of risk and vulnerability to climate change has been shown to motivate policymakers to implement adaptive responses (Birkland, 1997; O'Donovan, 2017), but also to create a path-dependency in future disaster response due to trauma that shapes heuristic responses to future disasters instead of considered responses that build broader adaptive capacity or resilience. For example, in Honduras, innovative disaster response policies implemented after Hurricane Mitch caused widespread devastation, dramatically improving disaster management, but climate policy response has since remained narrowly focused on disaster management and evacuation (Kuhl, 2021b). This kind of policy response to extensive climate disasters may lead to maladaptive responses (Felgenhauer, 2015), where the urgent need for disaster response and lack of long-term planning and stakeholder engagement can further entrench or even increase inequality and vulnerability to future disasters. Such a response may especially be the case for already-vulnerable populations (Atteridge and Remling, 2018), due to the shift in responsibility for disaster relief from the state to individuals or communities in the name of agency (Grove, 2014) or “self-reliance” (Joseph, 2013; Kuhl, 2021b; Welsh, 2014).

Researchers have found that policymakers may intentionally construct crisis declarations as tools for justifying and reinforcing political hegemony, especially in response to conflict and disasters (Armitage, 2002; Boin et al., 2009; Klein, 2015; Neocleous, 2006; Rivera, 2022). One danger is that policymakers may simply repackage existing policy agendas under the now-salient name of resilience and transformation (Ensor et al., 2021). Further, policymakers may be using resilience and transformation narratives to reinforce the status quo or push undemocratic change. Jurisdictions that directly experience disasters are more likely to have reactionary crisis responses that attempt to rebuild the status quo. Under these conditions, disasters and climate-related extreme events may ultimately inhibit transformational change. Other research contends that those indirectly impacted by disasters may take advantage of the policy window to deploy resources to build longer-term, transformational change (Friedman et al., 2019).

### 2.3. Managing trade-offs between resilience and transformation

**Fig. 1.**
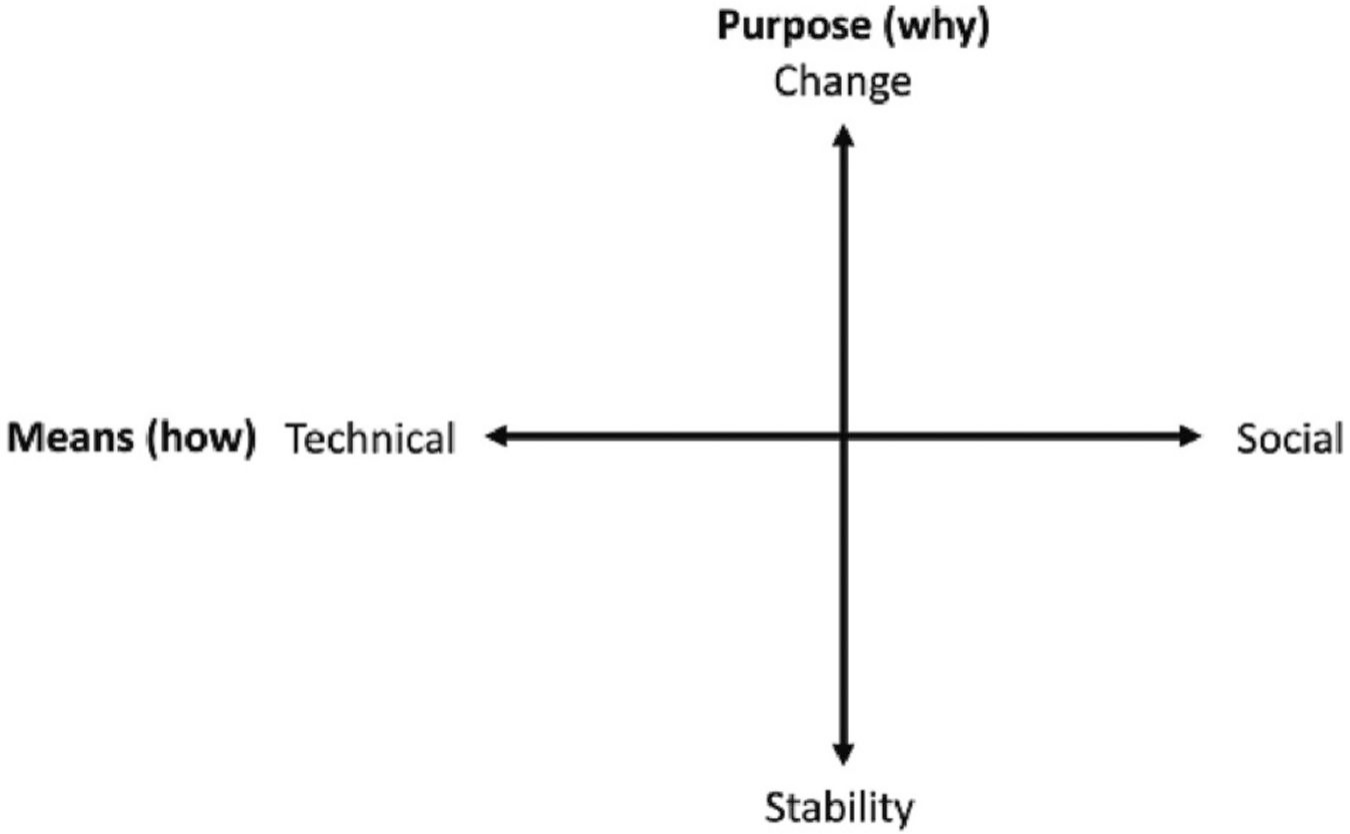
Trade-offs across resilience and transformation in climate and energy policies.

The contested conceptualization of resilience and its relationship to transformation can be employed by different actors to promote different visions of energy transitions, particularly in a post-disaster context. Essentially, actors strategically take advantage of inherent tensions within these concepts to promote different priorities. Based on this theoretical foundation, we present a conceptual framework that makes explicit tensions between competing priorities for resilience and transformation in climate and energy policy. Bi-directional tensions between resilience as stability or transformation as well as between emphases on technological or social priorities result in different policy priorities (Fig. 1). While policies can contain competing goals, the framework is intended to reveal the tensions that exist among these priorities and the trade-offs that policymakers must contend with when designing policy.

We suggest that these resilience and transformation policy trade-offs exist as two continua. Policies tend to be guided by particular visions of resilience and transformation that prioritize primary *objects* of transformation, *means* of attaining resilience, and *purposes* for pursuing these goals. One tension relates to the *purpose* of pursuing resilience and transformation, or the *why*; whether the objective is ultimately stability or change in systems. A stability-oriented emphasis in policy leads to the prioritization of maintaining existing infrastructure such as the centralized electric grid and institutions that profit from it at the expense of change-oriented reforms, such as toward more democratic governance of the energy system. The other tension relates to the *means* of achieving transformation or increased resilience, or the *how*. Climate and energy policies often emphasize building more resilient physical infrastructure through technological efforts such as installing renewable energy or building sea walls. Social infrastructure, including social networks, governance, and capital, however, are also vitally important for communities' resilience to catastrophes (Aldrich, 2018; Dilling et al., 2015; Eriksen et al., 2021; Folke et al., 2010; Gallopín, 2006; O'Brien et al., 2009). While energy resilience policies remain primarily oriented toward technology-based transitions to sustainable systems, there is momentum in climate governance discourse toward policies that build the social capacities to cope with disasters, adapt, or transform in their wake (Gallopín, 2006; Keck and Sakdapolrak, 2013; Smith and Stirling, 2010).

By setting the two tensions on axes, we show that policy priorities as suggested by their narratives can exist at any point along the two continua. Identifying the position of a narrative on each axis in this framework allows us to analyze the ways these two sets of priorities interact. Policymakers' decisions regarding why and how to achieve resilience and transformation in climate and energy decision-making have crucial implications for the role of resilience narratives in enabling or limiting transformations. In the rest of this paper, we apply this conceptual framework to our empirical analysis to characterize the types of narratives that have emerged in Puerto Rican climate and energy policy after Hurricane Maria.

## 3. Methods

### 3.1. Research context: climate and energy policy in Puerto Rico

Puerto Rico is a Caribbean archipelago that was colonized by the Spanish in 1493 and has been a territory of the United States since 1898 (De Onis, 2021). While Puerto Rico is officially bilingual in Spanish and English, Spanish is the primary language of the Commonwealth as a result of “decades of political struggle… and the extinction of the native language before [American] colonization, Taino” (Sotolongo et al., 2021). Climate and energy policy in Puerto Rico is strongly influenced by Puerto Rico's relationship with the United States. As a US territory, Puerto Rico is subject to US federal laws. However, unlike a U.S. state, Puerto Ricans do not have voting representation in the U.S. Congress or eligibility to vote in U.S. presidential elections (De Onis, 2021). At the territorial level, though, the policy process is similar to U.S. states, and Puerto Rico's legislative branch and executive agencies are responsible for climate and energy policy.

Puerto Rico is heavily dependent on fossil fuels; in 2022, 97% of the Commonwealth's electricity was generated by fossil fuel-fired plants: 43% fracked gas, 37% petroleum, and 17% coal (U.S. Energy Information Administration, 2023). Three percent of Puerto Rico's electricity was generated by renewable sources in 2021: primarily solar photo-voltaics with a small amount of wind, hydroelectric, and landfill gas generation. Puerto Rico faces energy distribution challenges as a result of the need to transmit energy from the site of the major power plants on the south coast of the main island to the majority of the population concentrated in the capital city, San Juan, as well as to many small communities that are relatively isolated and therefore difficult to access.

Financial issues in Puerto Rico's energy sector are longstanding. In recent history, the U.S. Congress responded to the Commonwealth's debt crisis, including the public utility's bankruptcy, by establishing a financial oversight board with the Puerto Rico Oversight, Management, and Economic Stability Act (PROMESA) in 2016. The combination of aging transmission and distribution infrastructure built in the 1950s, damage to this infrastructure by Hurricanes Irma and Maria in 2017, and damage to the two of the major power plants during the 2020 earthquakes have further contributed to substantial power interruptions and lack of reliability despite high energy costs to residents, issues that have culminated in the privatization of Puerto Rico's public utility, the Electric Power Authority (PREPA), in 2018 and the sale of its transmission and distribution system to LUMA Energy in 2020 (U.S. Energy Information Administration, 2023).

As evidenced by the significant service disruptions resulting from the 2017 and 2020 disasters, Puerto Rico's energy system is already vulnerable, but climate change, including more severe hurricane seasons, rising sea levels, and strains to the grid during extreme heat increase this vulnerability. These impacts will be experienced most intensely in the same marginalized communities that have historically faced environmental injustices across Puerto Rico. Scholars argue that the slow and insufficient response from the federal government to the hurricanes is the most recent example of a long colonial history of deprioritizing the survival of Puerto Ricans by dismissing requests for aid by Puerto Rico as another instance of “lazy Puerto Ricans waiting for handouts” (Rivera, 2022) and structurally embedding procedural injustices that exacerbate economic and environmental vulnerabilities despite Puerto Ricans' status as full U.S. citizens (Robinson et al., 2022). As evidenced by studies showing the negative health impacts and displacement of communities due to military weapons training, mining, hazardous waste at the former sites of pharmaceutical companies next to these communities, as well as the insufficient attention to these communities in disaster response efforts, vulnerability and environmental injustice are closely linked (De Onis, 2021; Lloréns, 2021; Santiago et al., 2020; Sotolongo et al., 2021).

### 3.2. Data

We examined a comprehensive set of Puerto Rican climate and energy policy documents pre- and post-Hurricane Maria (from 2008 to 2021). These were identified by searching online policy repositories hosted by the Puerto Rican Executive Departments of Agriculture, Natural & Environmental Resources, and Transportation & Public Works, the House of Representatives Commission of Agriculture, Natural Resources, & Environmental Issues, and the Senate Committees on Energy Matters, Innovation, Telecommunications, Urban Planning, and Environmental Health & Natural Resources. We screened all policies found on these websites for descriptions regarding environment, energy, or disaster management/recovery, and added policies that fit these criteria to a database of laws and policies for review. In total, we identified 235 Puerto Rican policies published between January 2008 and September 2021. We searched each policy for keywords we established a priori as potential synonyms to resilience, transition, and transformation in both Spanish and English (see Appendix A). Our dataset consisted of 86 relevant policies that included at least one keyword.

**Fig. 2.**
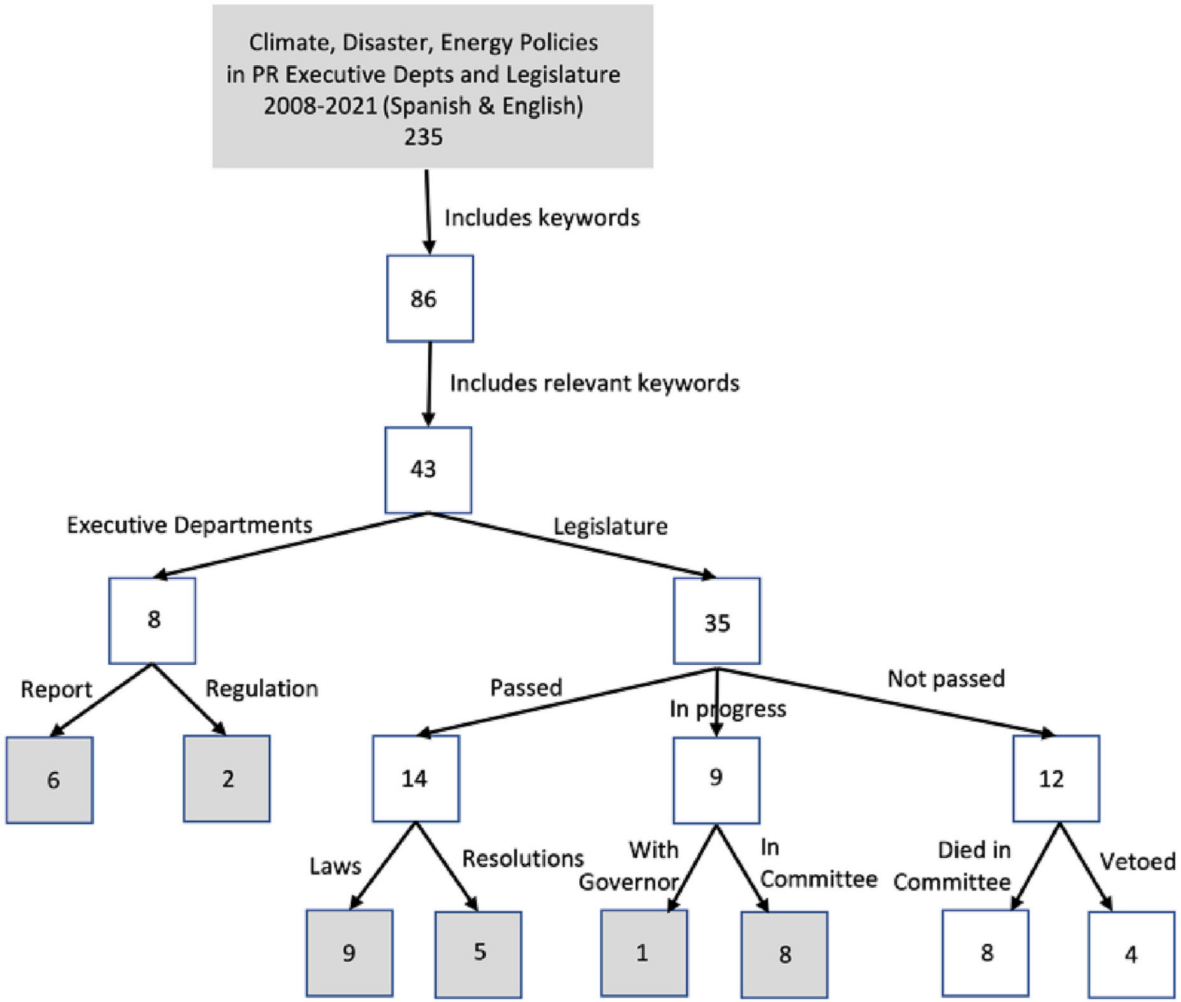
Policy document exclusion criteria.

After developing the dataset, we systematically examined each keyword in context through qualitative narrative analysis to determine relevance. Policies in which the keyword referenced the title of a different policy or was not relevant were excluded (i.e., transformation of cans in the recycling process). We also excluded policies that were vetoed, withdrawn, or not passed. Our final sample for analysis consisted of 31 policies (Fig. 2). This included two regulations from the Puerto Rico Energy Commission and Puerto Rico Energy Bureau, six reports from the Department of Natural and Environmental Resources, the Puerto Rico Resiliency Working Group, PREPA, the House Government Committee, and the Department of Housing. Nine were ratified laws, five were passed resolutions, and as of March 28, 2023, one bill was with the governor and eight bills were still in committee.

### 3.3. Analysis

We analyzed changes in conceptual framings of resilience and transformation over time using narrative synthesis, a systematic approach to comparative textual review and synthesis (Popay et al., 2006) that has been applied in climate and development research to analyze policies and interventions (Kuhl and Shinn, 2022; Snilstveit et al., 2012). First, we conducted quantitative content analysis by tabulating the frequency of each keyword across the policy dataset. We next manually inductively coded each occurrence of the keywords to identify themes of resilience and transformation with iteratively developed codes. Themes were organized into the *goals* for resilience and transformation as well as *how* resilience would be achieved (process) and *what* would be transformed (outcome). Text was coded with multiple themes depending on the level of detail. Some text did not include enough detail to be thematically coded (i.e., general references to resilience with insufficient detail to identify a goal or a process). Finally, we analyzed the frequency of the themes across policies and how these changed over time. We also considered the emphasis on the energy system as opposed to other sectors and the extent to which hurricanes or disasters were discussed in connection to resilience or transformation (Jones et al., 2020; O'Donovan, 2017). This allowed us to analyze how narratives of resilience and transformation changed in policy after the experience of Hurricane Maria.

The policy dataset was coded by two members of the research team, including one Spanish-speaker who reviewed and confirmed analysis. Discrepancies between coders were resolved through coordinated analysis. Because we identified relevant text through keywords, our analysis may have missed conceptualizations of resilience and transformation that did not explicitly use the keywords. However, we used a broad set of keywords in context as we reviewed each policy to mitigate this limitation. Given the importance of these terms in the policy narratives, it is quite unlikely that these concepts were discussed without reference to these terms or their synonyms. Recognizing that the policy process takes time, another potential limitation is that policies developed prior to Hurricane Maria may not have been proposed until after the hurricane, but these policies were likely to have been updated to reflect the recent catastrophe before being introduced, and thus the final versions likely did reflect the influence of Hurricane Maria in their framing. Finally, despite the presence of resilience and transformation narratives in policy documents, it is important to acknowledge that these narratives may not be reflected on-the-ground. Identifying policy goals is a different project from analyzing implemented actions.

## 4. Results

### 4.1. Policy impacts of Hurricane Maria

Our first objective was to analyze whether and how narratives of resilience and transformation changed after Hurricane Maria. Increases in the frequency of resilience and transformation-based references in policies after the hurricane, as well as references to Hurricane Maria or climate-induced disasters more generally, strongly suggest a connection between the impacts of the hurricane and the changing salience of transformation and resilience policy narratives.

There was a large increase in the number of policies that referenced resilience and transformation after Hurricane Maria. Table 1 lists the full set of relevant policies reviewed in this analysis. Before the hurricane, between 2008 and September 2017, 4 policies were passed that included resilience or transformation narratives (Fig. 2). The four policies that referenced transformation included an average of six relevant transformation keywords while the policy that referenced resilience included 14 relevant resilience keywords. After the hurricane, 27 policies were passed or remain on the legislative agenda between late 2017–2021, with an average of 5 relevant transformation keywords in the 22 policies referencing transformation and 13 relevant resilience keywords in the 22 policies referencing resilience.

Not only did the number of policies referencing resilience and transformation increase, but the narratives of resilience and transformation clearly illustrate the importance of the hurricane in shaping policy (Table 2). The one policy before Hurricane Maria that mentioned resilience described the importance of more resilient infrastructure, ecosystems, and communities for sustainable development. In the 22 policies in our dataset passed in the four years after Hurricane Maria, resilience was used primarily to identify the need for planning and technologies for grid resistance to disasters, with some emphasis on reliability and sustainability. In the four policies introduced prior to Hurricane Maria, transformation narratives primarily referenced a need for transformation of physical energy infrastructure for financial viability and economic development, indicating that while Hurricane Maria increased the salience of resilience of the energy system, these concerns did exist previously, and the issues were already present. Transformation narratives were included in 22 policies after Hurricane Maria (overlapping with the policies that included resilience, although some policies that included only one term or the other), most often framed as a need for regulation and energy infrastructure transformation to clean energy for economic benefit and resilience.

In addition to the dramatic increase in the prevalence of these terms after Hurricane Maria, references to the concept of disasters, Category 4 hurricanes, or Hurricane Maria were cited in 21 policies as part of calls for transformation (6) and resilience (18). Some of the policies, such as PS 1625–2020, specifically identified the destruction from Hurricanes Irma and Maria as a motivation and called for investment in hurricane recovery efforts, technologies, or agencies. Other policies referred to disasters more broadly to advocate for general policy change. Law 120–2018 was representative of narratives present across the portfolio:

With this Act, we begin the process to transform the Island's electric power system into a modern, sustainable, reliable, efficient, cost-effective [system] resilient to the ravages of nature. […] In addition, it must be a system resilient to weather events and the effects of climate change on the Island

(Law 120–2018, repeated in PS 424–2021).

Hurricane Maria left an indelible mark on climate and energy policy in Puerto Rico, most notably seen in the recurring objective of energy grid resistance to disasters.

### 4.2. Themes in narratives of resilience and transformation post-Hurricane

In this section we describe the themes that emerged regarding the means (how) and purpose (why) of increasing resilience, and the object (what) and purpose (why) of transformation.

#### 4.2.1. How to achieve resilience

Nineteen of the 27 policies included narratives of resilience achieved through planning. Multiple texts laid out the government's responsibility and intention to develop plans for resilience (Law 33–2019, BBR-2017, Law 72–2021) or initiate studies and reviews to improve resilience (IRP-2019), and highlighted the policies' role “*to set the parameters for a resilient, reliable, and robust energy system with just and reasonable rates*” (Law 17–2019). Although planning is a critical component of a response, many references were not particularly specific as to how this planning would lead to resilience.

The second most frequently referenced means to achieve resilience was through technology, which was included in 18 of the 27 policies. Most of these narratives framed resilience in terms of resilience of the physical power grid, which could be increased through technological innovation and adoption. There were frequent references to the promotion of clean energy technologies, particularly solar technologies and energy storage, as a means of creating resilience. Narratives of achieving resilience through technology, particularly for microgrids, were often connected to themes of reduced implementation costs and increased energy access, as demonstrated in NEPR-MI-2021-002, which considers whether MiniGrid construction is a “least cost” approach to resilience. These narratives highlight the interconnection between the *how* of achieving resilience through technology with the *why* of economic efficiency and cost-effectiveness. On the other hand, three policies combined themes of technological development with themes of sustainable development and wellbeing through democratization of energy systems: Law 258–2018, PREB 9117–2019, and PS 309–2021. This alternative narrative illustrates that it is possible to combine themes of achieving resilience through technology with multiple goals for resilience. However, despite the presence of multiple competing narratives regarding technology, the vast majority of policy narratives connected technological themes with economic themes.

Seven policies included narratives promoting resilience through infrastructure. Although it was not as common as the previous two themes, when policies did include this narrative, it was a dominant motivation. Focus on infrastructure often explicitly addressed the need for recovery of the physical electric grid and fortification against future storm damages. For example, the Action Plan for Disaster Recovery (APDR) argued that rather than repairing or replacing infrastructure, Puerto Rico needs to consider environmental changes to rebuild resiliently. Emphases on physical infrastructure for resilience were often accompanied by narratives that identified investments explicitly as the means through which this infrastructure resilience would be achieved, such as in Law 17–2019, which specified that “*capital investments are necessary to modernize and/or maintain in optimum conditions the Island's electric power grid in order to render it more reliable, resilient, and efficient”* (Law 17–2019).

Only two policies proposed economic development as a means to enhance resilience, directly connecting a lack of resilience to unemployment and inequality, including the APDR, which sets the objectives to: “*Develop a wide range of economic activities that increase the resilience of Puerto Rico, improve the skills and opportunities of existing employees, and reduce inequality*” (APDR-2018). Unlike narratives that connected resilience through technology with economic goals, here it was *through* economic development that resilience was achieved.

Approaches to resilience that did not focus on the energy system were rare in this dataset; one exception was an acknowledgement of the importance of ecosystem resilience in the APDR. Occasionally, resilience narratives were connected to public health, such as the explicit aim of Law 33–2019 to “*improve present and future public and environmental health”* in conjunction with sustainable development “*that shall make Puerto Rico more resilient”* (Law 33–2019). In contrast to the references to resilience that focused on energy systems, the level of detail in the narratives beyond energy was limited.

#### 4.2.2. Why increase resilience

The most common goal for resilience articulated in the policies was resistance to future threats, particularly disasters, present in 17 of the 27 policies. Concern for resilience to disasters reflected, as PS 1695–2020 put it, the *“magnitude of the damages caused by hurricanes Irma and Maria, the multiple recent earthquakes and the vulnerability of the Island to suffer more disasters”* (PS 1695–2020). Several policies underscored the importance of preparing for future threats by hardening the energy system: BBR-2017, ADPR-2018, Law 33–2019, IRP-2019, and PS 1695–2020. Hardening is a concept widely used in electrical engineering related to strengthening infrastructure as a means of increasing its ability to withstand threats. System hardening can be placed in contrast to other engineering strategies, most notably “smart” technologies (Panteli and Mancarella, 2015).

Goals of sustainable, clean energy and flexibility, modernization, reliability, and uninterruptedness of electricity were also common across policies. When sustainability was the goal for resilience, it was typically framed as a technological objective that contributed to Puerto Rico's broader goals of disaster resilience, as exemplified by the emphasis in RK 19–2021 placed on solar energy to address vulnerabilities revealed by disasters. These narratives also highlighted the goals' compatibility with requirements in existing energy policies like the Renewable Portfolio Standard. In addition to the technological frame, sustainability of the grid was frequently framed as an economic objective, as in the Integrated Resource Plan (IRP), where sustainability served the purpose of spurring economic growth and creating new markets. Flexibility, modernization, reliability, and uninterruptedness of the grid were often identified as lacking in the current system, thereby creating instability, as suggested in RKS 16–2021: “*Puerto Rico's electrical system lacks an orderly plan […] and integrated distributed generation and renewable energy sources that provide flexibility, reliability, resilience, and efficiency*” (RKS 16–2021). However, some resilience objectives were framed positively as aspirational goals of a future system. For example, Law 17–2019 stated that grid resilience contributes to sustainable development without further details on how.

Cost-effectiveness of the grid and resilience were connected in several policies including the IRP, reflecting the urgent need to address the debt burden of the energy system, high and unstable energy prices for end-users, and the costs of increasing grid resilience. Rather than reimagining the grid, here transformation referred to improving the existing infrastructure to lower debt and costs. Related to concerns regarding the cost-effectiveness of the grid but slightly less prevalent was the objective of reducing dependence on imported energy, as BBR-2017 described: “*Two use cases are proposed for [distributed energy resources] to build resilience for future emergencies and to reduce fossil fuel imports*” (BBR-2017). This suggests that goals of grid transformation included both increasing energy independence and decreasing fossil fuel consumption in Puerto Rico.

#### 4.2.3. What should be transformed

Infrastructure was the most commonly identified object needing transformation. Several references simply acknowledged the government's objective of energy infrastructure transformation, without providing any concrete details of what would be transformed or how this transformation would occur. Infrastructure was often discussed in conjunction with other objects of transformation, especially regulation, energy sources, and technology. The need for infrastructure transformation was also tightly coupled to disasters. For example, transformation narratives that specifically referred to disasters, as in RKS 19–2021, highlighted the importance of technological change in infrastructure systems to cleaner energy for transformation to a more sustainable and less vulnerable grid.

Transformation of regulation was the second most common theme, included in 14 policies. This also predominately emphasized energy and the grid, as suggested by policies with stated goals for energy regulatory frameworks such as in Law 120–2018, and occasionally financial objectives, as in the IRP, which stated for example: “*PREPA is working with the Government and its statutory fiscal agent, the Fiscal Agency and Financial Advisory Authority, to reach restructuring and the electric sector transformation*” (IRP-2019). As in this quote, these financial objectives tended to pertain to electricity via grid infrastructure or ratepayer costs.

After infrastructure and regulation, transformation of energy sources from fossil fuels to renewables was the most common theme in terms of what should be transformed. As this quote illustrates, there was recognition that the sources of energy needed to be transformed:

The power purchase contracts will be granted considering the goals and mandates established in the Renewable Energy Portfolio, which require a transition from power generation anchored in fossil fuels, to the aggressive integration of renewable energy

(PS 131–2021).

However, this quote also illustrates that this transformation was often characterized as being mandated or required externally.

Narratives concerning the transformation of institutional structures focused on privatization of the energy sector and presented privatization as a mechanism for achieving transformation as in RKS 16–2021, which noted the role of certain regulations in the increase in private participation and use of market signals for electric system transformation. Although privatization (or public-private partnerships) dominated the narratives of institutional change, other narratives of transformation through institutional structures discussed efforts to develop energy cooperatives, such as the objective highlighted in this regulation to integrate “*the Cooperative Movement by developing and consolidating the appropriate entities and by devising a common project that convenes the transformative actions of the cooperative movement to address the social and economic issues of Puerto Rico*” (PREB 9117–2019). These socially oriented narratives of transformation through cooperative governance aligned with narratives of transformation through distributed sources such as microgrids and community solar.

While most transformation narratives were focused on transformation of energy systems, there were three references to transformation of multiple systems including water, food, housing, and transportation after Hurricane Maria. Unlike the detailed focus on what transformation in the energy sector would consist of, these narratives combined different sectors into broad visions of transformation.

#### 4.2.4. Why transform

Sustainability was the most frequently cited motivation for why transformation should happen. Sustainability narratives sometimes identified environmental benefits, particularly when they were coincident with narratives of reducing fossil fuel dependence, as evident in this regulation: “*the Energy Bureau is mindful that Puerto Rico is in dire need of the transformation of its electric system to gain resiliency, improve environmental conditions and reduce Puerto Rico's dependance on fossil fuels*” (NEPR-MI-2021-002). Sustainability was often framed in terms of economic sustainability in addition to environmental sustainability, dual objectives the IRP illustrates:

to transform Puerto Rico's electric system into a modern and sustainable one, system ownership including generating assets will be open to private entities […]. The Governor of Puerto Rico has publicly stated that the reconstruction and transformation of the electricity sector will include the privatization of PREPA's generating facilities

(IRP-2019).

Although promotion of renewable energy was frequently identified as part of transformation processes, environmental sustainability rarely was upheld as its own objective, but rather as a co-benefit of the economic sustainability that renewable energy, particularly through privatization, would provide.

The second most common theme was growth, economic development, and prosperity through transformation of the economic system or economic development as evinced in Law 120–2018. Narratives of transformation for economic growth reframed Hurricane Maria as an opportunity for Puerto Rico to transform not only its electric system, but an opportunity to catalyze the investments in recovery and redevelopment needed to transform the economy of Puerto Rico. Although less common, three policies: Law 258–2018, PREB 9117–2019, and PS 309–2021, included a different conception of economic transformation by advocating for socioeconomic transformation through labor and energy cooperatives, with the goal of: “*transforming the popular mentality and making possible a new economic and social order*” as articulated in PS 309–2021, which would be achieved through education and economic opportunity.

The Build Back Better report exemplifies how, across the policies, resilience was identified as a goal of transformation: “*A transformed electric power system for Puerto Rico is one that is designed with the resiliency to withstand future storms and is built with modern grid technologies and control systems*” (BBB-2017). The policies that set resilience as the objective of transformation exemplified the tensions present in transformation and resilience more broadly between social and technological aims and stability and change.

Because of the fiscal crisis in Puerto Rico and bankruptcy of PREPA, it is not surprising that the goals of financial viability, cost-effectiveness, and debt reduction, found in eight policies, came through clearly in transformation narratives. These themes were related to economic growth and prosperity, but tended to be framed as less optimistic, forward-looking, and economy-wide. Instead, while sometimes positioned as ends of transformation, they were also framed as in tension with it, as in RS 270–2021, that established an investigative process to identify mechanisms to achieve:

the repayment of the debt of the Electric Power Authority (PREPA) that does not cause rate increases in the price of energy to the consumer, that are compatible with the mandates to reduce the cost of energy and transform the electrical system into one based on energy efficiency, renewable energy and energy resilience

(RS 270–2021).

While this narrative introduced multiple goals of transformation, it is also clear that debt repayment was the key priority, and all others can be interpreted as subsidiary.

Narratives of transformation toward modernization and efficiency, also found in eight policies, referred to efforts to reform the energy grid to be more reliable or stable through technology, as in Law 17–2019. Reducing energy costs was a parallel concern to cost-effectiveness on the consumer side, but this was not placed in tension with transformation. Rather, it was consistently an end goal and sometimes uncritically an assumed result of transformation such as through public-private partnerships in the energy grid as exemplified in Law 120–2018. In this policy, transforming the publicly owned utility to private sector ownership was presented as having the potential to achieve almost all of the goals for transformation identified across policies. While promising to deliver multiple transformation objectives, it was less clear how public-private partnerships will accomplish these goals.

## 5. Discussion

The proliferation of policies referencing resilience and transformation and their use of disaster terminology, including direct references to hurricanes after Hurricane Maria, demonstrates the rise of these concepts on the Puerto Rican policy agenda and the clear influence of Hurricane Maria on the narratives of resilience and transformation. Hurricane Maria opened a window of opportunity for climate and energy policy that drew policymakers' attention to existing, underlying climate and disaster-relevant vulnerabilities and presented an opportunity to craft new policy in response to this need.

The literature on policy change suggests that in the event of crises such as extreme weather events, there will be an increased focus on the systemic insufficiencies that caused the crisis or exacerbated its impacts, and to which policymakers may respond (Birkland, 1998; Kingdon, 1984; Rudel, 2019). Policymakers may perceive this attention as an opportunity to use the emergency context and the vulnerabilities it underscores to introduce and implement recovery plans as well as future disaster risk reduction more successfully, including through building physically and socially resilient systems. The large number of new policies drawing on resilience and transformation suggests that Hurricane Maria did increase the salience of these terms, but at the same time, when analyzing the goals and means through which resilience and transformation would be achieved, it was less clear that Hurricane Maria shifted the overarching direction of Puerto Rican climate and energy policy.

This analysis also revealed that narratives of resilience and transformation were used to advance objectives of both stability and change in the energy system. There were clear tensions in the narratives between pursuing stability, i.e., through reforms that make the existing centralized grid more reliable and resistant to disasters, and change, i.e., through reforms that advance energy democracy and decentralization. However, the majority of uses of both key terms referred to technologically enabling physical electric grid infrastructure reliability, especially in the face of future disasters. In many cases, even when transformation was evoked, it was in service of changes needed to promote the stability of the existing system. This suggests that after Hurricane Maria, achieving stability was the dominant resilience and transformation goal.

It was in the narratives of how to advance resilience and transformation that our results notably deviated from the tensions identified in the literature as shown in Fig. 1. In our analysis, we found that economic narratives, which were central to both how and why resilience and transformation would be achieved, did not fit neatly into this continuum. Economic narratives connected stability in physical infrastructure to economic growth and privatization of energy systems. While finance has the capacity to support both social and technological innovation, in the policy narratives it was more closely aligned with technological goals and tended to appear alongside technology-based narratives of creating a more reliable grid. Alternative narratives, such as those focusing on justice or equity as drivers or results of resilience and transformation did not emerge from our analysis because, despite a few instances of the terms, these concepts were not predominant themes compared to economic, technological, and environmental sustainability considerations.

In 2020, the Canadian-American LUMA Energy power company, which specializes in fracked gas infrastructure, won a contract to rebuild, maintain, and modernize Puerto Rico's energy infrastructure by pledging that as a private company, it could better rebuild infrastructure and handle hurricanes (Mazzei, 2021). Despite this, more than five years after the hurricane, Puerto Ricans continue to face higher electricity costs than most US states, as well as ongoing disruptive and harmful blackouts (De Onis, 2021). In March 2022, Puerto Rico's public utility, PREPA, stated it did not believe it was possible to meet its commitment under the 2019 Puerto Rico Energy Public Policy Act to achieve 40% renewable energy by 2025, even though a major 2021 study found that building decentralized rooftop solar could generate 75% of Puerto Rico's electricity by 2035 (Biaggi et al., 2021; Tigue, 2022). This is not surprising given that post-hurricane infrastructure efforts led by LUMA Energy have focused on fracked gas rather than community-based energy or social resilience initiatives. None of the power system repair and upgrade projects approved by the Puerto Rico Energy Bureau to PREPA's 2010 10-year Infrastructure Plan included renewable energy infrastructure (Puerto Rico Energy Bureau, 2022; Tigue, 2022). Future climate-induced disasters are likely to exacerbate the power grid's limitations (Mazzei, 2021). As long as the entities responsible for the Puerto Rican energy system emphasize stability without addressing longer-term transformational change, residents of Puerto Rico are unlikely to experience a sustainable energy transition.

## 6. Conclusions

Our analysis revealed the breadth of conceptualizations of both resilience and transformation in Puerto Rican climate and energy policy, suggesting that these concepts can be used to promote different visions of sustainable transitions, particularly in a post-disaster context. Most evident was the fact that resilience and transformation narratives almost exclusively centered the energy system as their object, as opposed to other potential sectors where goals of resilience and transformation are also relevant.

We further found that in a post-disaster context, transformation narratives were employed to support efforts that increased the stability of vulnerable existing systems. Across policies, we found evidence of varied, sometimes conflicting narratives of resilience and transformation. The policies conveyed competing narratives of resilience and transformation, and identified a wide range of technological and social visions that can support change to the energy system, such as microgrids and labor and energy cooperatives. However, while these concepts clearly became more salient after a climate-induced disaster, narratives of stability were dominant – in this case, a focus on updating and maintaining the electric system –while policies encouraging transformational change beyond the existing system remained limited. Overall, the narrative analysis showed an emphasis on reducing the vulnerability of existing physical systems, often through technology and privatization, instead of enabling changes to address the social inequities that underlie the energy system and reinforce harms caused by energy disruptions.

Ultimately, this analysis identified that the policy narratives promoted preservation rather than innovation. The pursuit of stability constrained what types of technology and social change were possible to implement in practice. A policy approach overly focused on maintenance and reform of existing systems may reflect a coping approach to disaster recovery and energy transition rather than broader multi-systemic transformation. Such approaches align more with what scholars have characterized as a reactive pathway that is more likely to lead to maladaptive response rather than a proactive pathway to transformation (Novalia and Malekpour, 2020).

While we do not suggest that the Puerto Rican experience is generalizable, or fixed, as Puerto Rico's energy future is actively debated and being developed, if the experience in Puerto Rico is indicative of the policy response in other contexts, it suggests that the desire for stability may inhibit sustainability transitions after a disaster. Given the calls for urgent transformations of energy systems and increasing rate and intensity of the climate crisis, greater attention to the role of disasters and the ways that narratives of transformation may support stability of existing systems is needed in the literature. As the climate crisis grows, research evaluating the effects of climate-induced extreme events on policymaking is increasingly important for understanding and shaping the policy process and recognizing both the potential and limitations for sustainability transitions.

## Figures and Tables

**Table 1:** Puerto Rican (PR) climate and energy policies.

Name of Document (Document Type and Number-Year Introduced)	Date	Description	Keyword Counts	
			Resilience	Transformation
PR Energy Transformation and RELIEF Act (Law 57–2014)	5/27/2014	To reform PREPA to serve the energy needs of the public including creating an independent Energy Commission for greater oversight, and the Commonwealth Energy Public Policy Office to develop energy public policy.	0	6
Electric Power Authority Revitalization Act (Law 4–2016)	2/1/2016	To reform PREPA to (1) reduce debt; (2) reform governance; (3) implement operations savings; (4) promote public-private investment; (5) maintain reasonable and accessible rates; and (6) comply with federal and state regulations.	0	15
The Adaptation Plan for Climate Change (CC Report - 2016)	3/1/2016	To study of the vulnerability of public infrastructure to climate change (CC).	14	1
Joint Senate Resolution 0059 (RCS 59–2017)	2/6/2017	To order PREPA and the Public Partnership Authority to assess a proposed gas power generation plant and the Public Private Partnership model.	0	2
Hurricane Maria Resolution Activating the Committee for the Adoption of Building Codes in Response to Hurricane Maria (RS 172–2017)	11/7/2017	To order the Executive Director of the Permit Management Office to activate the Committee for the Adoption of Building Codes to update the PR Building Code, in light of the effects of Hurricane Maria on the island's infrastructure.	1	0
Report: Build Back Better: Reimagining and Strengthening the Power Grid of PR (BBR-2017)	12/1/2017	To outline the damage inflicted by Hurricane Maria and propose recommendations for a reimagining of the power grid.	34	3
Regulation on Microgrid Development of the PR Energy Commission (CEPR-MI-2018-0001)	5/1/2018	To promote microgrid development to deliver reliable energy services, avoid the loss of power, promote consumer choice, reduce carbon pollution, and spur economic development with new technology.	1	0
PR Electric Power System *Transformation Act (Law 120–2018)	6/20/2018	To identify that PREPA does not effectively serve the people of PR and enable public-private partnerships for energy system transformation.	5	21
PR Energy Cooperatives Law (Law 258–2018)	9/1/2018	To establish a public policy related to energy cooperatives in the PR energy model.	2	3
Report: PR Action Plan for Disaster Recovery (APDR –2018)	11/1/2018	To consider how to meet urgent humanitarian needs with investments in transformative recovery.	85	18
Report: Investigation into Nuclear Power Plants in PR (RC 1189–2018)	11/1/2018	To order an investigation into establishing nuclear power plants in PR to produce energy.	0	2
Law Declaring CC Education Day (Law 152–2019)	12/7/2018	To declare October 24 of each year as the Day of Education on Climate Change in PR.	3	0
Law Ordering School Solar Feasibility Study (Law 75–2018)	1/17/2019	To order an investigation into the feasibility of installing solar or other renewables in schools for clean energy and cost-effectiveness.	0	1
PR Integrated Resource Plan 2018–19 (IRP-2019)	2/1/2019	To consider reasonable resources to satisfy demand for electrical services over twenty years.	72	14
Regulation on Electric Cooperatives in PR (PREB 9117–2019)	3/1/2019	To prescribe the regulatory framework for electric cooperatives.	3	5
Resolution Ordering CDBG-DR Funds to the Department of Transportation and Public Works (RCS 368–2019)	4/1/2019	To order the Department of Housing of PR to manage Community Development Block Grants - Disaster Recovery (CDBG-DR) and enable amendments for the use of CDBG-DR funds in response to Hurricanes Irma and Maria.	0	1
PR Energy Public Policy Act (Law 17–2019)	4/11/2019	To establish parameters for a resilient, reliable, and robust energy system with just, reasonable rates.	25	17
PR CC Mitigation, Adaptation, and Resilience Act (Law 33–2019)	5/22/2019	To set forth the public policy of the Government of PR on climate change and on the mitigation, adaptation, and resilience processes per sector.	22	8
Law to Give Executive Oversight to Private Performance in the Electric System* (PS 1693–2020)	12/17/2020	To give the executive branch greater oversight over the performance of the private sector in PR's electrical system.	1	0
Law Creating the COR3 of PR* (PS 1695–2020/ PC 2626–2020)	12/21/2020	To create the Central Office for Recovery, Reconstruction and Resilience of PR (COR3) to supervise compliance with federal disaster grants and coordinate with municipalities.	6	2
Law Amending the Mitigation, Adaptation, and Resilience Act* (PS 84–2021)	1/2/2021	To amend the Law of MAR to CC in PR, allocating funds to the committee of experts and advisors on CC to comply with the law's objectives, and preparation of the plan for MAR.	0	1
Law for the elimination of the combustion of coal in the generation of energy in PR* (PS 131–2021)	1/2/2021	To create a law eliminating the combustion of coal in the generation of energy in PR.	1	1
Report: Determination on alignment with the Approved IRP and Modified Action Plan (NEPR-MI-2021-002)	1/25/2021	This IRP filing is the second PREPA IRP proceeding and follows the IRP approved in 2015, which included significant findings and directives regarding the acquisition, retirement, and development of additional resources by PREPA.	4	2
Law to Establish Energy Labor Cooperatives* (PS 309–2021)	4/19/2021	To establish energy labor cooperatives.	2	1
Law to Require Solar Installation in all new Buildings* (PC 783–2021)	5/10/2021	To make solar installation compulsory in new buildings and mandate that all new buildings provide sufficient strength and size roofs for solar installations.	1	1
Law specifying PREPA employee rights* (PS 424–2021)	5/13/2021	To specify the rights of electric power authority employees.	1	4
Resolution on report on LUMA Public-Private Partnership (RKS 16–2021)	6/1/2021	Resolution on the report on action relating to the public-private partnership with LUMA Energy.	1	5
Law designating a Hurricane Education Season (PS 87–2021)	6/24/2021	To designate May–July pre-Hurricane educational season to promote the prevention, awareness, strengthening, and empowerment of PR citizens	1	0
Resolution to reject the electric transition charge* (RKS 19–2021)	6/30/2021	To reject the transition charge on electric bills, including the charge to self-generation through renewable energy.	3	1
Resolution to investigate PREPA debt payment mechanisms without raising energy costs* (RS 270–2021)	6/30/2021	To order an investigative process to identify mechanisms to repay PREPA's debt without raising energy costs and maintain compatibility with mandates to transform the system to one based on energy efficiency, renewables, and resilience.	5	3
Resolution to reject electricity rate increases due to LUMA* (RCC 228–2021)	9/24/2021	To reject an increase in electricity rate due to unreasonable or inefficient actions on the part of LUMA Energy.	0	1

* Indicates policies that have not (yet) been passed.

**Table 2:** Transformation and resilience frames in PR climate and energy policies.

Concept	Narrative Frames in Policies	Pre- Hurricane Policy Count	Post- Hurricane Policy Count
Resilience	How: planning	1	18
	How: technology (including microgrids)	1	17
	How: infrastructure	1	7
	How: non-energy systems (including ecosystems and communities)	1	6
	How: investment	0	5
	How: economic development	0	2
	Why: resistance to threats and system hardening	1	16
	Why: sustainability and clean energy	0	13
	Why: flexibility, modernization, reliability, and uninterruptedness	0	11
	Why: cost-effectiveness	0	9
	Why: decreased dependency	0	6
	Why: sustainable development and well-being	1	5
	Why: reconstruction	0	5
Transformation	What: infrastructure	3	15
	What: regulation	2	12
	What: energy sources	2	11
	What: institutional structures	1	9
	What: technology (including microgrids)	0	6
	What: non-energy systems (including ecosystems, water, housing)	1	3
	Why: clean energy and sustainability	1	14
	Why: growth, economic development, and prosperity	2	9
	Why: resilience	0	9
	Why: financial viability, cost-effectiveness, debt reduction	2	8
	Why: modernization and efficiency	1	8
	Why: fossil fuel dependence reduction	1	7
	Why: energy cost reduction	0	6
**Total Policy Count**		**5**	**27**

## Data Availability

The data is publicly available

## Appendix A. List of English and Spanish keywords related to resilience and transformation used to search Puerto Rican climate and energy policy

- Resistencia
- Resilience
- Resiliency
- Resiliencia
- Resiliente
- Transformation
- Transform
- Transformed
- Transforming
- Transition
- Transformacion
- Transicion
- Transformar
- Transformando
